# Audiologic Assessment and Management of Teprotumumab-Associated Ototoxicity: An Updated Narrative Review

**DOI:** 10.3390/audiolres16030092

**Published:** 2026-06-19

**Authors:** John Williams, Alex Elkins, Alp Sarigul, Mary Frances Johnson, Charles E. Bishop

**Affiliations:** 1 School of Graduate Studies in the Health Sciences, University of Mississippi Medical Center, Jackson, MS 39216, USA; jwilliams32@umc.edu (J.W.);; 2 Department of Otolaryngology—Head and Neck Surgery, University of Mississippi Medical Center, Jackson, MS 39216, USA; mfjohnson@umc.edu; 3 School of Medicine, University of Mississippi Medical Center, Jackson, MS 39216, USA

**Keywords:** teprotumumab, thyroid eye disease, ototoxicity, audiologic monitoring, sensorineural hearing loss, eustachian tube dysfunction

## Abstract

**Introduction:** Teprotumumab (Tepezza^®^), an insulin-like growth factor-1 receptor (IGF-1R) antagonist, is the first FDA-approved targeted therapy for thyroid eye disease (TED). While effective for reducing proptosis and inflammation, increasing post-marketing evidence has linked teprotumumab to auditory adverse events. IGF-1 signaling is essential for cochlear maintenance and neuroprotection; therefore, systemic IGF-1R inhibition presents a biologically plausible mechanism for ototoxicity. Despite growing recognition of these effects, no standardized approach exists for audiologic assessment or monitoring of patients receiving teprotumumab. This review aimed to (1) summarize proposed mechanisms and the reported spectrum of teprotumumab-related auditory effects, (2) evaluate current methods used to assess and monitor these patients, and (3) identify areas of consensus and ongoing uncertainty. **Methods:** An updated narrative review of the literature was conducting using PubMed, CINAHL, and Google Scholar using Boolean strings targeting teprotumumab exposure and hearing-related outcomes. Studies from 2022 onward were identified using Boolean search strings targeting teprotumumab exposure and hearing-related outcomes. Peer-reviewed English language studies reporting audiometric findings were eligible for inclusion. **Results:** Ten studies met inclusion criteria. Reported effects most commonly included bilateral high-frequency SNHL, tinnitus, and aural fullness, typically emerging after three to six infusions. Many cases demonstrated persistent deficits despite drug discontinuation. Baseline audiometric assessment was not uniformly reported across studies, and monitoring protocols varied considerably, with inconsistent incorporation of speech testing and immittance measures. **Conclusions:** Teprotumumab-associated ototoxicity is increasingly recognized and potentially irreversible. Current evidence is insufficient to guide standardized monitoring. Prospective studies are urgently needed to establish evidence-based audiologic surveillance protocols.

## 1. Introduction

Thyroid eye disease (TED) is an autoimmune inflammatory disorder characterized by orbital fibroblast activation, leading to tissue expansion and progressive remodeling of both adipose tissue and extraocular muscles. These pathologic changes result in the pathognomonic manifestations of TED, including protruding eyes, double vision, and eyelid retraction. The underlying pathophysiology of TED is now understood to involve a complex interaction between the thyroid-stimulating hormone receptor (TSHR) and the insulin-like growth factor-1 receptor (IGF-1R) expressed on orbital fibroblasts. Overactivation of this TSHR/IGF-1R signaling complex promotes the release of pro-inflammatory cytokines, accumulation of glycosaminoglycan, and differentiation of orbital fibroblasts, all of which ultimately drive orbital inflammation, connective tissue remodeling, and retrobulbar adiposity [1].

Teprotumumab (Tepezza^®^) is a fully human monoclonal antibody that acts selectively as an IGF-1R antagonist and represents the first FDA-approved targeted medical therapy for TED. By inhibiting IGF-1R signaling, teprotumumab disrupts a critical upstream pathway in TED pathogenesis, significantly reducing orbital inflammation, eye protrusion, and double vision. Clinical trials demonstrated substantial improvements in vision-related clinical activity scores and patient-reported outcomes, establishing teprotumumab as a highly effective therapy for moderate-to-severe or recalcitrant TED [2,3,4]. However, post-approval experience has increasingly revealed adverse effect profiles that were not fully appreciated during early clinical trials, particularly with respect to otologic toxicity [5].

The biologic rationale for teprotumumab-associated ototoxicity stems from the essential role of IGF-1 signaling in the auditory system. IGF-1 and IGF-1R are widely expressed within the cochlea, where they contribute to hair cell survival, synaptic maintenance, and neuroprotection [6,7]. Experimental models have demonstrated that disruption of IGF-1 signaling accelerates age-related hearing loss and increases susceptibility to noise-induced and metabolic cochlear injury [7]. Inhibition of IGF-1R through systemic therapies therefore carries a plausible risk of cochlear toxicity. Further, teprotumumab’s tissue remodeling effects have been hypothesized to induce atrophy of nasopharyngeal fat pads, such as Ostmann’s fat pad, increasing the risk of middle ear issues, including Eustachian tube dysfunction [8]. Together, these mechanisms suggest that teprotumumab may produce both cochlear and middle-ear auditory sequelae.

Growing clinical evidence supports this mechanistic concern. Reported otologic manifestations associated with teprotumumab include tinnitus, hypoacusis, autophony, sound distortion, hyperacusis, aural fullness, and clinically significant hearing loss [9,10]. Among these, high-frequency sensorineural hearing loss (SNHL) has been the most consistently documented audiologic abnormality [8,11]. Importantly, early clinical trials did not require baseline or follow-up audiometric testing and relied primarily on patient self-report, likely underestimating both the frequency and severity of these adverse events [3,4].

One of the earliest and most influential publications drawing attention to this issue was the multi-institutional case series by Belinsky and colleagues [8]. In this report, the authors described four patients who developed audiometrically confirmed hearing loss during teprotumumab therapy, ranging from mild high-frequency deficits to severe, persistent bilateral SNHL [8]. Notably, one patient was referred for a cochlear implant due to significant decreases in hearing acuity and speech perception. Belinsky and colleagues emphasized that auditory symptoms did not reliably resolve following drug discontinuation and that no standardized approach to monitoring existed. In response, they proposed a formal surveillance protocol including baseline audiometry, interval testing during treatment, and post-therapy follow-up—representing the first structured recommendation for audiologic monitoring in this population. Shortly thereafter, Chow and Silkiss published a detailed case report further highlighting the potential for chronic, irreversible teprotumumab-associated hearing loss [12]. Their patient developed bilateral sensorineural loss after five infusions, with no audiometric improvement six weeks after therapy cessation. The authors recommended even more rigorous screening, advocating for audiometric testing before treatment and at regular intervals throughout the infusion course. Together, Belinsky and Chow issued parallel calls for action, emphasizing that early identification and longitudinal audiologic monitoring are essential to appropriately assess and manage teprotumumab-related hearing toxicity.

Since these calls to action, a rapidly expanding body of literature has confirmed that audiologic adverse events occur more frequently than originally appreciated. Systematic reviews and meta-analyses have suggested that teprotumumab-associated ototoxic events may affect a clinically significant proportion of treated patients [13,14]. Pooled analyses have estimated that objective audiometric threshold elevations only occur in approximately 40% of patients with available pre- and post-treatment testing [15]. Pharmacovigilance studies using the FDA Adverse Event Reporting System (FAERS) have similarly identified hearing loss as one of the most commonly reported serious adverse events associated with teprotumumab, with reported incidences ranging from approximately 13–16% in large real-world datasets [16,17,18]. Observational cohort studies have reported even higher rates, in some series exceeding 20% [19]. Taken together, these findings suggest that the true prevalence of teprotumumab-associated ototoxicity was likely underestimated in early randomized trials.

Despite increasing recognition of this complication, substantial gaps remain in clinical guidance. At present, there is no widely accepted consensus regarding how patients on teprotumumab should be screened, monitored, or managed from an audiologic standpoint. Published studies vary considerably in methodology: many lack baseline audiometry, rely on subjective symptom reporting, or provide limited characterization of hearing outcomes [4,10]. The absence of standardized definitions for clinically significant threshold shifts, optimal testing intervals, or management algorithms has resulted in highly variable clinical practice patterns across institutions [5,8]. Furthermore, risk factors for developing ototoxicity—including age, baseline hearing status, noise exposure history, and cumulative dosing—remain poorly defined [20,21].

Although several reviews have addressed teprotumumab safety more broadly, there has not yet been an updated synthesis focused specifically on audiologic assessment and management in patients receiving this therapy, despite known ototoxic consequences. The foundational recommendations offered by Belinsky and Chow were published in 2022 and necessarily relied on limited early experience [8,12]. Since that time, a substantial volume of additional evidence has emerged, including reports of persistent hearing loss, tinnitus, and other otologic sequelae that may substantially affect quality of life. Despite growing recognition of these complications, there remains no broadly accepted evidence-based framework for audiologic monitoring, risk stratification, or management of patients receiving teprotumumab. An updated evaluation integrating this newer literature is therefore critically needed.

The aim of the present narrative review is to evaluate current evidence regarding audiologic dysfunction associated with teprotumumab therapy and to summarize existing approaches to patient assessment and management. To our knowledge, there has not yet been an updated review focused specifically on the audiologic evaluation and management of patients treated with teprotumumab that incorporates the rapidly expanding post-2022 literature. Specifically, we seek to (1) review the proposed mechanisms and reported spectrum of teprotumumab-related auditory effects, (2) examine the methods currently used to assess and monitor these patients in clinical practice, and (3) highlight areas of consensus and ongoing uncertainty.

## 2. Methods

A literature search was conducted in PubMed, Cumulative Index to Nursing and Allied Health Literature (CINAHL), and Google Scholar to identify studies evaluating audiologic outcomes in patients treated with teprotumumab (Tepezza^®^). This study was conducted as a narrative review rather than a formal systematic review. Accordingly, the search strategy was designed to provide a broad overview of the available literature and identify key studies relevant to audiologic outcomes associated with teprotumumab. Searches were performed using appropriate, predefined Boolean search strings targeting teprotumumab exposure and hearing-related outcomes (Table 1). Duplicates were removed and abstracts were screened for relevance by at least two authors to control for bias. In order to highlight updated literature, database filters were applied to include studies published from 2022 onward. A PRISMA-style flow diagram was generated with the assistance from OpenAI ChatGPT (GPT-5.5, OpenAI, San Francisco, CA, USA) based on author-determined study selection counts and was reviewed and edited by the authors for accuracy prior to submission. The study selection process, inclusion and exclusion decisions, data extraction, interpretation, and manuscript content were determined and verified exclusively by the authors.

Studies were deemed eligible for inclusion if they were peer-reviewed, written in English, and reported results from audiological testing in patients who received teprotumumab therapy. Eligible designs included clinical trials, cohort studies, case series, and case reports. Studies were excluded if they were non-peer-reviewed, including conference abstracts, published prior to 2022, did not include audiometric assessment, were not available in English, or were not accessible in full text. Non-eligible studies included press releases and conference abstracts. Titles and abstracts were screened for relevance, followed by full-text review of potentially eligible articles. Data were extracted on study design, patient characteristics, audiologic testing methods, and reported hearing-related outcomes.

## 3. Results

**Figure 1:**
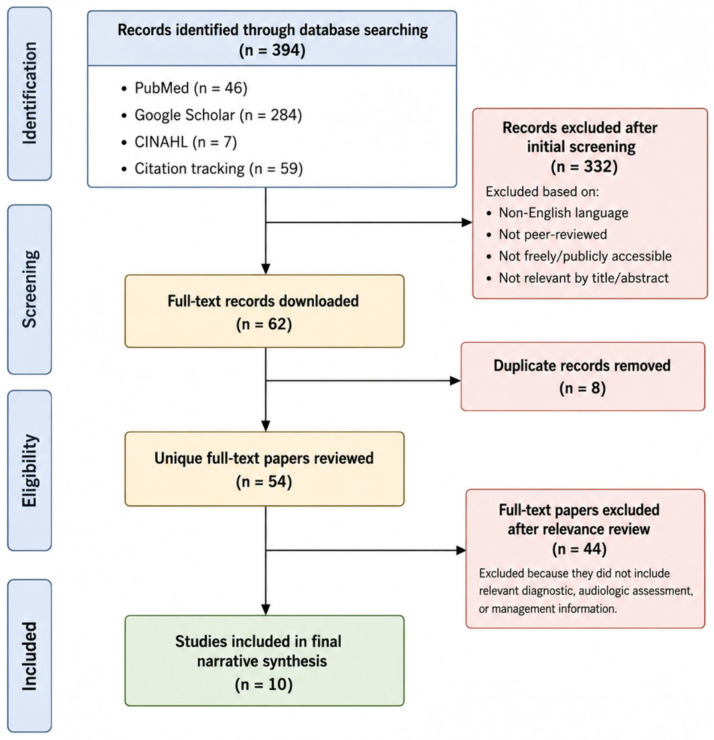
PRISMA-style flow diagram of study selection. A total of 394 records were identified through database searching and citation tracking. After screening and duplicate removal, 54 unique full-text articles were reviewed for eligibility. Forty-four articles were excluded following full-text review, leaving 10 studies for inclusion in the final narrative synthesis.

Initial searches identified *n* = 394 records, including *n* = 46 from PubMed, *n* = 284 from Google Scholar, *n* = 7 from CINAHL, and *n* = 59 through citation tracking. After initial screening for English language, peer-review status, free/public full-text availability, and title/abstract relevance, *n* = 332 records were excluded, leaving *n* = 62 full-text articles for download and further review. Eight duplicates were removed, resulting in *n* = 54 unique full-text articles assessed for eligibility. Following full-text review, *n* = 44 articles were excluded because they did not provide relevant diagnostic, audiological assessment, or management information. Ten studies met inclusion and were included in the final narrative synthesis (Figure 1). Included studies can be seen in Table 2.

### 3.1. Proposed Mechanisms and Reported Spectrum of Auditory Effects

Across all studies, teprotumumab-associated ototoxicity most commonly manifested as bilateral high-frequency sensorineural hearing loss (SNHL), frequently accompanied by tinnitus, aural fullness, sound distortion, and difficulty understanding speech in noise. Case reports consistently demonstrated measurable declines in pure-tone thresholds during therapy. Ding et al. described a patient who developed moderate-to-severe bilateral SNHL with markedly reduced word recognition scores following infusion six, resulting in significant functional impairment [11]. Similarly, Highland et al. reported progressive symmetric SNHL with persistent deficits despite attempted corticosteroid therapy [9].

Self-reported symptoms and audiometric data do not always align. Keen et al. presented an analysis of twenty-two patients (e.g., forty-four ears) who received teprotumumab infusions, comparing thresholds before, during, and after treatment [25]. The study reported large percentage—nearly 40% of the ears included in analysis—of patients developed a confirmed hearing loss that met ototoxicity criteria. Notably, approximately half of the patients with a confirmed hearing loss reported no symptoms; conversely, some individuals with a perceived hearing change did not demonstrate audiometric threshold shifts.

The timing of symptom onset was relatively consistent across studies, typically occurring mid-treatment between the third and sixth infusions [9,10,11,20]. This temporal pattern suggests a possible cumulative dose-related effect rather than an acute idiosyncratic reaction.

While cochlear toxicity was the dominant pattern, several reports highlighted symptoms suggestive of middle-ear involvement, including autophony and aural fullness [23,24]. Kay-Rivest et al. specifically described multiple patients meeting clinical criteria for patulous Eustachian tube (PET), raising the possibility that teprotumumab may also alter Eustachian tube function through tissue remodeling effects [24]. Hsiou et al. reported a patient with persistent bothersome aural fullness in the absence of measurable audiometric hearing loss; notably, immittance measures and tests of middle ear function—including assessing for PET—were not completed [23]. These findings support the hypothesis that teprotumumab-associated ototoxicity may involve both sensorineural and conductive mechanisms, consistent with the known biologic role of IGF-1R signaling in cochlear maintenance and nasopharyngeal tissue integrity.

Evidence regarding reversibility was mixed but overall concerning. Multiple studies reported persistent hearing loss despite discontinuation of therapy [9,11,20]. However, Lu et al. described a patient whose early-identified mixed hearing loss improved following prompt initiation of oral corticosteroids, suggesting that early recognition may influence outcomes [26]. Additionally, Phansalkar et al. demonstrated that resuming therapy at a half-dose regimen stabilized previously observed threshold shifts, implying that dose modification may mitigate further toxicity [27].

### 3.2. Methods Used to Assess and Monitor Patients

Assessment methods ranged from minimal testing to comprehensive ototoxicity batteries. Components of these test batteries varied but most commonly included conventional pure-tone audiometry (250–8000 Hz), with some studies incorporating extended high frequency (>8000 Hz), speech audiometry, tympanometry, and distortion product otoacoustic emissions (DPOAEs). Some cohorts employed only screening-level audiometry, while others used full diagnostic protocols including conventional audiometry, extended high-frequency audiometry, tympanometry, speech discrimination testing, and DPOAEs [24].

The presence—or lack thereof—of baseline audiometry emerged as one of the most critical methodological variables. Among included studies, the proportion of patients with documented pre-treatment audiometry varied, with several case reports lacking baseline data completely. Najjar and colleagues were able to clearly document objective threshold shifts due to available pre-treatment testing, whereas Hsiou et al. demonstrated the difficulty of interpreting post-treatment results without a baseline reference [20,23].

Monitoring intervals also varied widely. Some studies assessed hearing only after the development of subjective symptoms, whereas others incorporated prospective pre- and post-treatment testing [9,22,24]. Douglas et al. conducted a prospective study with scheduled testing and found that 50% of patients with pre-existing hearing loss experienced further decline, highlighting the potential value of structured surveillance [22]. When reported, follow-up assessments most commonly occurred either after completion of the infusion course or following the onset of new otologic symptoms, with fewer studies employing predefined interval testing during therapy.

Importantly, many studies relied solely on pure-tone thresholds, limiting the ability to differentiate cochlear toxicity from Eustachian tube dysfunction. Speech audiometry, immittance testing, and objective cochlear measures (e.g., DPOAEs) were inconsistently reported across studies and were not routinely included as part of the test battery.

## 4. Discussion

The purpose of this review was to (1) summarize the proposed mechanisms and clinical spectrum of teprotumumab-associated ototoxicity, (2) examine how patients are currently assessed and monitored in clinical practice, and (3) identify areas of consensus and persistent uncertainty. Across ten recent studies incorporating objective audiometric testing, consistent patterns emerged. Teprotumumab therapy is associated with a range of otologic adverse effects, most commonly high-frequency sensorineural hearing loss, tinnitus, and aural fullness. Symptoms tend to develop after several infusions, often around the third to sixth cycle, and may persist despite discontinuation of therapy. Collectively, the results demonstrate that teprotumumab-associated ototoxicity is a real and clinically meaningful phenomenon—but also reveal that current clinical practices are insufficient to adequately address it.

An additional layer of complexity is the frequent mismatch between symptoms and audiometric findings. Keen et al. demonstrated that a large proportion of patients with a significant threshold shift did not report symptoms, while symptomatic individuals showed no measurable change [25]. This discordance may, in part, reflect variations amongst how “ototoxicity” was defined across studies. Existing classification systems vary considerably, generally falling into three categories: change-from-baseline criteria (e.g., ASHA), absolute-threshold classification (e.g., Brock, SIOP), and hybrid systems incorporating both threshold shifts and functional impacts (e.g., NCI-CTCAE) [28]. While change-based criteria are sensitive for early cochlear insults, subclinical changes that do not subsequently correspond to noticeable functional deficits may be identified. Conversely, absolute-threshold systems better identify perceived functional deficits, but may fail to capture early ototoxic effects. Hybrid systems attempt to balance these approaches.

### 4.1. Lack of Standardization of Audiologic Monitoring

The findings summarized under Aim 3 underscore a central and troubling conclusion: at present, audiologists and other clinicians do not have enough standardized clinical information to appropriately monitor patients receiving Tepezza^®^. Keen et al. explicitly emphasized that “…no universal protocol exists for audiometric testing in patients starting teprotumumab,” reflecting the lack of standardization across clinical settings [25]. Despite growing recognition of otologic risk, there remains no universally accepted protocol regarding who should be tested, what tests should be performed, how frequently they should occur, or how to respond when changes are detected.

### 4.2. Variability in Assessment Methods & Baseline Data

Some studies relied solely on pure-tone screening audiometry, while others employed comprehensive batteries including extended high-frequency testing, speech measures, tympanometry, and otoacoustic emissions. Several reports evaluated patients only after subjective complaints arose, whereas others incorporated prospective monitoring. This lack of uniformity prevents reliable comparison across studies and limits clinicians’ ability to interpret hearing changes in individual patients. Baseline audiometric assessment was not uniformly reported across studies, limiting interpretation of treatment-related hearing changes and complicating comparisons across cohorts. Consequently, the true incidence, severity, and reversibility of teprotumumab-associated ototoxicity remain difficult to establish.

### 4.3. Limitations of Current Audiologic Test Batteries

Another major area of uncertainty concerns which audiologic tests are most appropriate for monitoring. Pure-tone thresholds alone may be insufficient to capture the full spectrum of teprotumumab-related effects. Several studies reported prominent complaints of aural fullness, autophony, and sound distortion—symptoms suggestive of middle-ear or Eustachian tube dysfunction. However, immittance testing was rarely performed, and speech audiometry was inconsistently reported. Kay-Rivest et al. demonstrated that more comprehensive protocols including extended high-frequency audiometry and DPOAEs may detect changes missed by conventional testing, yet such approaches are not routinely used in clinical settings [24]. As a result, clinicians lack a universally accepted, evidence-based framework regarding which components of an audiologic battery are truly necessary.

Indeed, an important and likely under-recognized aspect of teprotumumab-associated ototoxicity is the potential for middle-ear involvement, particularly Eustachian tube dysfunction. While most studies emphasize sensorineural mechanisms, Kay-Rivest et al. and Hsiou et al. highlight symptoms such as aural fullness and autophony, which are symptoms consistent with Eustachian tube dysfunction, sometimes occurring in the absence of measurable hearing loss [23,24]. These observations suggest that teprotumumab-associated toxicity may not be exclusively cochlear and may reflect parallel conductive or middle-ear processes. From a clinical standpoint, these findings underscore the potential limitations of relying solely on pure-tone audiometry when evaluating patients receiving teprotumumab. Incorporation of immittance testing, including tympanometry and assessment for Eustachian tube dysfunction, may provide critical diagnostic information.

### 4.4. Reversibility and Treatment Considerations

The question of reversibility further complicates clinical decision-making. Several studies reported persistent hearing loss despite drug discontinuation, and corticosteroid therapy was generally ineffective when administered after deficits were well-established. However, the case described by Lu et al. suggests that very early identification and prompt intervention may improve outcomes [26]. Similarly, Phansalkar et al. provided preliminary evidence that dose reduction rather than full discontinuation might allow continued treatment without further auditory decline [27]. These isolated reports are encouraging but insufficient to inform routine practice. At present, clinicians have no reliable algorithm to determine when therapy should be paused, modified, or stopped in response to hearing changes.

### 4.5. Risk Factors and Patient Vulnerability

Although identification of patient-level risk factors was not a primary objective of the present review, several studies raised important observations that may help guide future investigations and clinical monitoring. Among currently proposed risk factors, pre-existing hearing loss is supported by the strongest available evidence. Sears et al. identified baseline hearing loss a significant predictor of teprotumumab-related ototoxicity, while Douglas et al. observed a greater likelihood of hearing decline and persistent deficits among patients with abnormal baseline audiometry [10,22]. Collectively, these findings suggest that patients with pre-existing auditory dysfunction may represent a particularly vulnerable subgroup and support the use of baseline audiometric assessment prior to treatment initiation.

In contrast, several additional risk factors remain speculative. Individual reports have suggested possible associations with cumulative treatment exposure, age-related susceptibility, metabolic comorbidities, and underlying thyroid disease characteristics. However, these observations have not been consistently replicated across studies and currently lack sufficient evidence to support risk stratification.

### 4.6. Vestibular Considerations

While outside the scope of the present review, the authors acknowledge that the possibility of vestibulotoxicity should not be ruled out. “Dizziness”—which is, by nature, a broadly defined symptom—has been measured subjectively in clinical trial data and post-marketing surveillance data. Indeed, Epperson and colleagues measured subjective dizziness via the Dizziness Handicap Inventory (DHI) and found that individuals with teprotumumab-associated hearing changes more frequently reported dizziness [29]. While the exact mechanisms are currently unknown, previous studies have suggested that IGF-1 plays a protective role against vestibular hair cells, much like in the cochlea [30].

### 4.7. Implications for Clinical Practice

Taken together, these gaps reveal a stark reality: audiologists are currently being asked to monitor patients on a potentially ototoxic medication without adequate tools, standards, or guidelines. Unlike established ototoxic agents such as cisplatin or aminoglycosides—where decades of research have produced clear monitoring protocols—teprotumumab has entered clinical practice without an accompanying framework for auditory safety surveillance. The result is a patchwork of inconsistent practices that may delay detection of preventable harm.

Based on the available evidence, a pragmatic monitoring approach can be proposed until formal consensus guidelines are established). Prior to initiation of teprotumumab therapy, patients should undergo a comprehensive audiological assessment to characterize baseline function, including otoscopy, tympanometry, pure-tone audiometry, and speech testing. Patients should be counseled regarding potential auditory and otologic symptoms, including hearing loss, tinnitus, aural fullness, and autophony. During treatment, audiologic evaluations should be completed every 2–3 infusions, or sooner if patients report new or worsening audiologic symptoms. At minimum, serial testing should include pure-tone and speech recognition measures, with tympanometry when symptoms suggest middle-ear or Eustachian tube involvement. Following completion of therapy, post-treatment surveillance should be conducted to document recovery, stability, or progression or auditory changes and to guide further management.

In addition to establishing a standardized testing schedule, a framework for classifying treatment-related hearing changes is needed. Among currently available ototoxicity grading systems, a hybrid approach incorporating both ASHA threshold-shift criteria and NCI Common Terminology Criteria for Adverse Events (CTCAE) may be most appropriate for patients receiving teprotumumab. ASHA criteria provide a sensitive and widely accepted method for detecting early audiometric changes, whereas CTCAE grading incorporates symptom severity and functional impact. This distinction, where many patients frequently report tinnitus, aural fullness, autophony, and subjective hearing difficulties that may not correlate directly with pure-tone threshold changes. Conversely, isolated use of pediatric oncology-focused grading systems such as Brock or SIOP may inadequately capture the diverse auditory manifestations reported in this population. Until teprotumumab-specific criteria are developed and validated, the combined use of ASHA criteria for ototoxicity detection and CTCAE grading for severity of classification may provide the most clinically meaningful framework for monitoring and reporting treatment-related auditory adverse events.

This situation has important implications for interdisciplinary care. Teprotumumab is prescribed primarily by ophthalmologists and endocrinologists, yet the burden of monitoring falls largely on audiologists and otolaryngologists. Effective management therefore requires coordinated, team-based protocols that currently do not exist. Without agreed-upon testing schedules, referral triggers, and decision pathways, patients may experience avoidable and potentially irreversible hearing loss.

### 4.8. Limitations of the Present Review

This review has several limitations that should be considered when interpreting its findings. First and foremost, this review was not conducted as a formal, PRISMA-guided systematic review or meta-analysis; although a structured search strategy and predefined inclusion criteria were used, study selection and synthesis were not performed using a standardized systematic review framework. As a result, the possibility of selection bias and incomplete capture of all relevant studies cannot be excluded. Second, the available literature on teprotumumab-associated ototoxicity remains limited and is composed primarily of small cohort studies and case reports, many of which lack standardized audiologic assessment protocols. This introduces substantial heterogeneity in study design, patient populations, and outcome measures.

Baseline audiometric data were inconsistently obtained across studies, limiting the ability to definitively attribute observed hearing changes to teprotumumab exposure and complicating cross-study comparisons. Additionally, a lack of standardization in defining “ototoxicity”—including differences in threshold-based versus symptom-based criteria—further challenges interpretation and synthesis of results. Lastly, the retrospective nature of several included studies and the absence of long-term follow-up data limit conclusions regarding the reversibility and clinical significance of these auditory effects. Collectively, these limitations underscore the need for prospective studies employing standardized methodologies, comprehensive audiologic test batteries, and uniform definitions of ototoxicity.

## 5. Conclusions

Teprotumumab therapy for thyroid eye disease is increasingly recognized as a potential cause for clinically significant auditory dysfunction, most commonly high-frequency sensorineural hearing loss. Although the biologic plausibility of IGF-1R-mediated ototoxicity is well-supported, current evidence remains limited by small study sizes and methodological heterogeneity. Available data suggest that auditory deficits may persist despite treatment discontinuation, underscoring the importance of early recognition and audiologic surveillance. Prospective studies employing standardized methodologies are needed to better characterize incidence, risk factors, and long-term outcomes.

## Figures and Tables

**Table 1:** Search strategies included Boolean strings searched in PubMed, Google Scholar, and EBSCO/CINAHL.

Search Strategy	
PubMed	(tepezza[tiab] OR teprotumumab[tiab]) AND (“Hearing Loss”[Mesh] OR hearing loss[tiab] OR hypoacusis[tiab] OR tinnitus[tiab] OR “patulous eustachian tube”[tiab] OR “eustachian tube dysfunction”[tiab])
Google Scholar	(“tepezza” OR “teprotumumab”) (“hearing loss” OR tinnitus OR “eustachian tube dysfunction”)
CINAHL	(Tepezza OR teprotumumab) AND (hearing loss OR hypoacusis OR tinnitus OR “patulous eustachian tube” OR “eustachian tube dysfunction” OR PET OR ETD)

**Table 2:** Summary of study characteristics, audiologic assessment methods, symptom onset, baseline hearing status and reported reversibility of symptoms across included studies. Conventional audiometry refers to standard pure-tone and speech audiometric testing. Cohort studies that included participants with both normal and abnormal baseline hearing were classified as “Mixed Cohorts” in the baseline hearing status column.

	Study Design	Cohort Size	Baseline Hearing Status	Audiometry	ME Testing	DPOAEs	Symptom Onset	Reversibility?
[11]	Case Report	*N* = 1	Not Completed	Conventional	No	No	Subjective @ 3–4; confirmed after 7	Persistent
[9]	Case Report	*N* = 1	Not Completed	Conventional	No	No	After 5; worsened after 5–7	Persistent
[22]	Prospective observational Study	*N* = 52	Mixed Cohorts	Screening Audiometry	No	No	---	Partial recovery common, persistent deficits remained in a subset at 6 months
[23]	Case Report	*N* = 1	Not Completed	Conventional	No	No	3; DC’d therapy after 4	Aural fullness resolved by 8 months
[24]	Prospective Cohort	*N* = 35; *N* = 14 with pre/post audiometry	Mixed Cohorts	Conventional + UHF	Tympanometry and PET	OAEs	After 2–4	Post-treatment audiometric changes remained detectable; reversibility not systematically addressed
[25]	Retrospective Case Series	*N* = 22 (*N* = 44 ears)	Mixed Cohorts	Conventional, No Speech	No	No	After 2–4	4/8 symptomatic patients with ≥3-month f/u reported no improvement
[26]	Case Report	*N* = 1	Not Completed	Conventional	No	No	10 days after 1	Reversible following oral prednisone
[20]	Case Report	*N* = 1	Clearly Normal	Conventional	No	No	Tinnitus after 2; hearing loss after 5	No improvement 1-momth following cessation
[27]	Case Report	*N* = 1	Clearly Normal	Conventional	No	No	After 4	Partial improvement following cessation of full-dose therapy
[10]	Prospective observational case series	*N* = 27; *N* = 6 with pre/post audiometry	Mixed Cohorts	Conventional, Speech Not Reported	PET	No	After 3.8	45.5% of subjective hearing loss symptoms resolved; persistent audiometrically confirmed SNHL in 3/5 patients

UHF = Ultra-high Frequency; PET = Patulous Eustachian Tube; DC = Discontinued; ME = Middle Ear; DC = Discontinued; F/U = Follow-Up; SNHL = Sensorineural Hearing Loss; OAEs = Otoacoustic Emissions.

## Data Availability

No new data were created or analyzed in this study.
